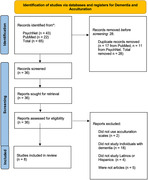# Acculturation scales in Dementia in US Latinos/Hispanics: A Systematic Review

**DOI:** 10.1002/alz.093426

**Published:** 2025-01-09

**Authors:** Alicia Goytizolo, Monica Rosselli

**Affiliations:** ^1^ Florida Atlantic University, Davie, FL USA; ^2^ 1Florida Alzheimer’s Disease Research Center, Miami, FL USA

## Abstract

**Background:**

Acculturation is a relevant variable in the assessment of dementia. Little is known about the type of acculturation scales and the frequency in which they are used in dementia research among Latinos/Hispanics in the US. The purpose of this paper was to conduct a systematic review of published studies that investigated the use of acculturation measures toward Latinos and Hispanics who are diagnosed with dementia.

**Methods:**

Databases were searched using four key search terms: dementia, acculturation scale, Latinos, and Hispanics. We followed the Preferred Reporting Items for Systematic Reviews and Meta‐Analyses (PRISMA) criteria, which included: a sample population of Latinos/Hispanics who were diagnosed with dementia; and the use of an acculturation scale as part of their methods. Review articles, book chapters, and dissertations were excluded. The methodology and findings of all retrieved articles were thoroughly evaluated to determine eligibility. Data were extracted from all eligible articles regarding the study population, study methods, outcomes measured, acculturation measures used, and results.

**Results:**

The literature search using PubMed and PsychNET databases disclosed sixty‐five articles that were originally reviewed. Twenty‐eight articles were excluded as a result of not meeting the criteria. A total of eight articles were included in the current analysis with a total combined sample of 1323 individuals (Mean age = 67.5, SD; 8.39* female percentage = 0.68). Participants were recruited from California (4 studies), Texas (2 studies), New York (2 studies), Puerto Rico (1 study), Florida (1 study), Illinois (1 study), and Pennsylvania (1 study). The use of five different acculturation scales were identified (Marin Acculturation Scale, Short Acculturation Scale for Hispanics, Bidimensional Acculturation Scale for Hispanics, Abbreviated Multidimensional Acculturation Scale, and the Acculturation Rating Scale for Mexican Americans‐II). 50% of the reviewed articles found a significant association between acculturation and cognition.

**Conclusions:**

The scales and measurements being used to determine the influence of acculturation on dementia in the Latino and Hispanic population continue to be under researched. The limited number of published studies on the topic results in an inability to generalize the conclusions on a larger scale.

*Excludes one data set due to unavailable data